# Daily sedative interruption versus intermittent sedation in mechanically ventilated critically ill patients: a randomized trial

**DOI:** 10.1186/2110-5820-4-14

**Published:** 2014-05-06

**Authors:** Antonio Paulo Nassar Junior, Marcelo Park

**Affiliations:** 1 Hospital das Clínicas, Universidade de São Paulo, Rua Dr Enéas de Carvalho Aguiar, 255, Fifth Floor, Emergency Medicine Discipline, São Paulo 05403-000, Brazil

**Keywords:** Sedation, Mechanical ventilation, Conscious sedation, Critical care and outcome assessment

## Abstract

**Background:**

Daily sedative interruption and intermittent sedation are effective in abbreviating the time on mechanical ventilation. Whether one is superior to the other has not yet been determined. Our aim was to compare daily interruption and intermittent sedation during the mechanical ventilation period in a low nurse staffing ICU.

**Methods:**

Adult patients expected to need mechanical ventilation for more than 24 hours were randomly assigned, in a single center, either to daily interruption of continuous sedative and opioid infusion or to intermittent sedation. In both cases, our goal was to maintain a Sedation Agitation Scale (SAS) level of 3 or 4; that is patients should be calm, easily arousable or awakened with verbal stimuli or gentle shaking. Primary outcome was ventilator-free days in 28 days. Secondary outcomes were ICU and hospital mortality, incidence of delirium, nurse workload, self-extubation and psychological distress six months after ICU discharge.

**Results:**

A total of 60 patients were included. There were no differences in the ventilator-free days in 28 days between daily interruption and intermittent sedation (median: 24 versus 25 days, *P* = 0.160). There were also no differences in ICU mortality (40 versus 23.3%, *P* = 0.165), hospital mortality (43.3 versus 30%, *P* = 0.284), incidence of delirium (30 versus 40%, *P* = 0.472), self-extubation (3.3 versus 6.7%, *P* = 0.514), and psychological stress six months after ICU discharge. Also, the nurse workload was not different between groups, but it was reduced on day 5 compared to day 1 in both groups (Nurse Activity Score (NAS) in the intermittent sedation group was 54 on day 1 versus 39 on day 5, *P* < 0.001; NAS in daily interruption group was 53 on day 1 versus 38 on day 5, *P* < 0.001). Fentanyl and midazolam total dosages per patient were higher in the daily interruption group. The tidal volume was higher in the intermittent sedation group during the first five days of ICU stay.

**Conclusions:**

There was no difference in the number of ventilator-free days in 28 days between both groups. Intermittent sedation was associated with lower sedative and opioid doses.

**Trial registration:**

ClinicalTrials.gov Identifier: NCT00824239.

## Background

Sedation is an important component for the care of critically ill patients who require mechanical ventilation. However, it is commonly overused [1], and, when in excess, it is associated with increased time on mechanical ventilation [2], higher mortality [3], delirium [4] and psychological disturbances [5]. Fortunately, there are some strategies which have been shown to be effective in reducing the burden of excessive sedation.

Daily interruption of sedative infusions is the most well-studied approach. Its use has been shown to shorten duration of mechanical ventilation and ICU stay [6,7]. Nursing-implemented sedation protocols are also associated with decreased days on mechanical ventilation [8]. Recently, a protocol of no sedation has shown reduced duration of mechanical ventilation compared to daily interruption of sedative infusion, without severe adverse events [9]. However, all trials that showed the efficacy of lighter levels of sedation were conducted in developed countries, which have a higher nursing staff level than developing countries [10]. Lighter sedation strategies in a lower nursing staff level ICU may expose patients on mechanical ventilation to care-associated risks, such as accidental extubation, since there is a trend between increased nurse staffing levels and decreased adverse patient outcomes in ICU [11].

In short, since 2006, our local practice has been to keep our patients on mechanical ventilation without sedation, prioritizing optimized analgesia and allowing them to express pain and collaborate in physical therapy. With the hypothesis of a safe mechanical ventilation time reduction using less sedation, we conducted a randomized, controlled trial to compare two strategies that allow patients to be awake - intermittent sedation, as we had adopted, or daily interruption of sedative infusion - aiming to compare the groups based on the number of ventilator-free days in a 28-day period and on safety issues in a developing country ICU.

## Methods

### Design and setting

This trial was conducted in a closed multidisciplinary six-bed ICU that admits patients from emergency department, surgical room and ward in an academic tertiary hospital from January 2009 to December 2011. The ICU has one intensive care physician, one intensive care resident and three second-year internal medicine residents on daytime shifts. During the night, there is one intensive care physician and one second-year internal medicine resident on charge. This intensive care physician is also responsible for another 14-bed ICU, where an intensive care resident and a first-year internal medicine resident are on charge. The nurse-to-patient ratio is 1:6 and the nursing assistant-to-patient ratio is 1:2 on all shifts. Nursing assistants are only responsible for the patients’ hygiene and administration of drugs in peripheral venous and nasoenteral catheters. There is one respiratory and physical therapist only on daytime shifts.

### Patients

Eligible patients were those who required mechanical ventilation within the last 24 hours and were expected to need mechanical ventilation for more than 24 hours. Patients were excluded if they were younger than 18 years-old, were pregnant, needed deep levels of sedation (intracranial hypertension, status epilepticus, hypothermia after cardiac arrest, severe asthma exacerbations, and severe hypoxemic respiratory failure (PaO_2_/FiO_2_ ratio < 50), were not expected to survive for more than six months (for example, metastatic cancer, NY functional class IV heart failure, Child C hepatic cirrhosis, oxygen-dependent chronic obstructive pulmonary disease), were previously cognitive impaired (for example, advanced dementia), or were readmitted to the ICU after participating in the trial.

### Randomization

Research staff randomized included patients in a 1:1 ratio to intermittent sedation or continuous infusion of sedatives with daily interruptions. Treatment allocation was concealed by random selection of opaque sealed envelopes for consecutive patients from a box with 120 envelops. Every envelope contained a paper in which the sedation strategy of that patient would be allocated. The study was unblinded. Therefore, attending clinicians and research staff were aware of which group the included patients were allocated to.

### Ethics statement

The study (protocol number 0284/08) was approved by the Ethical Committee for Research Projects Analysis of Hospital das Clínicas, University of São Paulo, in September 2008. Written informed consent was obtained from patients or their legal representatives. If consent was given by a representative, patients were contacted for their consent as soon as possible.

### Procedures

All patients had their level of sedation monitored with a Portuguese version of Sedation Agitation Score (SAS) [12] every six hours by bedside nurses. Sedation was defined as the infusion of sedative drugs, that is, midazolam or propofol, at the discretion of the attending physician. Patients in the intermittent sedation group would be kept without continuous infusion of sedatives if the intubation had been performed in the ICU, or would have their infusion interrupted after randomization if they had been admitted already intubated from emergency department, surgical room, wards or another ICU. Patients would be kept without sedatives infusion until they awoke. After patients were awake, if they were calm and collaborative (SAS of 4), they would be kept without infusion of sedatives. If the patient was uncomfortable or agitated (SAS ≥ 5), the physician (attending or resident) would be consulted, and possible causes of discomfort would be investigated in a standardized method (pain, patient-ventilator asynchrony, thirst, hunger, and position on the bed, all of them using a poster which included figures expressing these uncomfortable sensations) and treated. Pain was treated with boluses of fentanyl (50 to 150 μg). If the pain recrudesced in less than two hours or there was a persistent pain stimulus (for example, surgical scar, drains) a continuous infusion of fentanyl would be initiated and titrated by the attending nurse using numeric pain scale (which measures pain from 0 = no pain, to 10 = the worst pain ever experienced) aiming a value ≤ 4. If agitation had no visible cause and pain was already empirically treated with a bolus of fentanyl, then delirium would be suspected and haloperidol administrated (bolus of 2.5 or 5 mg). After 15 minutes, if the patient was still uncomfortable or agitated, a continuous infusion of midazolam or propofol would be initiated to achieve a SAS of 3 to 4. The choice between midazolam and propofol was at the discretion of the attending physician. Sedative dosing would be titrated every two hours thereafter or sooner if the patient was agitated (that is, SAS ≥ 5). Interruption of sedatives infusions would then be performed during the next shift (morning, afternoon or night) in order to try to keep the patient without sedation again.

The daily interruption group would receive sedatives (midazolam or propofol, at the discretion of the attending physician) aimed to a SAS target of 3 to 4, which means that the patient is awake or easily arousable with verbal or gentile physical stimulus. SAS would be recorded at least three times a day. Every morning, after changing shifts (7 am), sedative and opioid infusions would be interrupted by bedside nurses until patients were awake and could follow simple commands (open their eyes, look at the clinician, squeeze the hand, open their mouth). The sedative infusion would be restarted at half the previous dose only if the patient was agitated (SAS ≥ 5). If the patient could not follow commands after sedative interruption because of agitation, the infusion would also be restarted at half the previous dose and titrated to a SAS of 3 to 4, if possible. Sedative infusions would only be titrated during that day if the patient was agitated (SAS ≥ 5). Pain was evaluated with the numeric scale every two hours. It was treated the same way as in the intermittent sedation group. In case of suspected delirium, that is, agitation with no visible cause after the administration of fentanyl, haloperidol would be administrated as described above.

All patients had their sedative infusions interrupted when positive end-expiratory pressure (PEEP) was set at 5 cmH_2_O and inspiratory fraction of oxygen ≤ 0.4, when a spontaneous breathing trial (T-tube trial) would be performed if the patient was hemodynamically stable (mean arterial pressure ≥ 65 mmHg, no substantial use of norepinephrine or dobutamine, respiratory rate < 35 per minute, PaO_2_/FiO_2_ ratio > 150). If the patient was re-intubated, he or she would be treated again according to the previously randomized group protocol.

Baseline demographic data, Acute Physiology and Chronic Health Evaluation (APACHE II) score [13], the reason for admission and for intubation were recorded for all patients. Minimal and maximal heart rate, mean arterial pressure and respiratory rate were recorded from day 1 to day 5 or extubation, whichever came first. Dosage of inotropic, vasopressors and daily fluid balance were also recorded. Ventilation variables such as ventilator mode, peak pressure, driving pressure, PEEP, tidal volume, P/F ratio and arterial pressure of carbon dioxide (PaCO_2_) were also recorded from day 1 to 5. Delirium was assessed by the internal medicine resident on charge twice daily using Confusion Assessment Method for ICU (CAM-ICU) [14], also on the first seven days or until ICU discharge. Nurse workload was evaluated daily until day 5 with the Portuguese version of Nursing Activity Score (NAS) in morning shifts [15]. Total sequentialorgan failure assessment (SOFA) scores were also calculated daily until day 5 [16]. All patients who survived until hospital discharge were contacted after six months and evaluated for the level of psychological stress using the Impact of Event Scale (IES) [17].

### Outcomes

The primary study outcome was ventilator-free days in 28 days. Secondary outcomes were ICU and hospital mortality, ICU and hospital length-of-stay, incidence of delirium, delirium and coma-free days in seven days, percentage of time on target SAS, self-extubation and reintubation, accidental removal of catheters, tracheostomy rates, total sedatives doses per patient, differences in hemodynamic and ventilator variables, SOFA score, nurse workload and level of psychological stress six months after ICU discharge.

### Statistical analysis

We calculated that to provide 90% power with *P*-values of less than 0.05, a sample size of 106 patients would be required to detect a mean difference of 2 days of mechanical ventilation with a standard-deviation of 3.15 days in favor of the intermittent sedation approach. We intended to accomplish two interim analyzes after 35 and 70 patients, in order to evaluate the safety of the interventions (that is, self-extubations and accidental removal of catheters). We postulated that a difference in adverse events with a significance level *P* < 0.01 in these analyses would preclude the trial from recruiting new patients. Nevertheless, since the previewed period of enrollment ended, and only 60 patients were included, a new sample size analysis was conducted using the current data. Moreover, a sample size of 170 patients would be necessary to reach the programmed difference in mechanical ventilation-free days within 28 days. At this moment, based on the slow recruitment rate, the decision to stop the trial was made.

Data were analyzed on an intention-to-treat basis. Continuous data is presented as median (percentile 25th, percentile 75th) and compared with the Mann-Whitney *U*-test. Categorical data are presented as absolute values and percentages, and compared with chi-square or Fischer’s exact tests as appropriate. Interaction analyses were performed using a mixed generalized model with the patient as random factor in order to account for the within-subject correlation among repeated observations. The Markov Chain Monte Carlo procedure with 1,000 simulations to reach the equilibrium of distributions was used to retrieve a fixed probability of each resulting independent variable. The *post hoc* analyses for interactions were performed using the Mann-Whitney or Wilcoxon’s tests as appropriated. Kaplan-Meier survival analysis was used to assess the effects of the sedation strategy on the duration of mechanical ventilation, while log-rank tests were used to examine differences in survival curves. The R free source statistical package was used to build the graphics and to perform all the statistical analyses.

## Results

**Figure 1:**
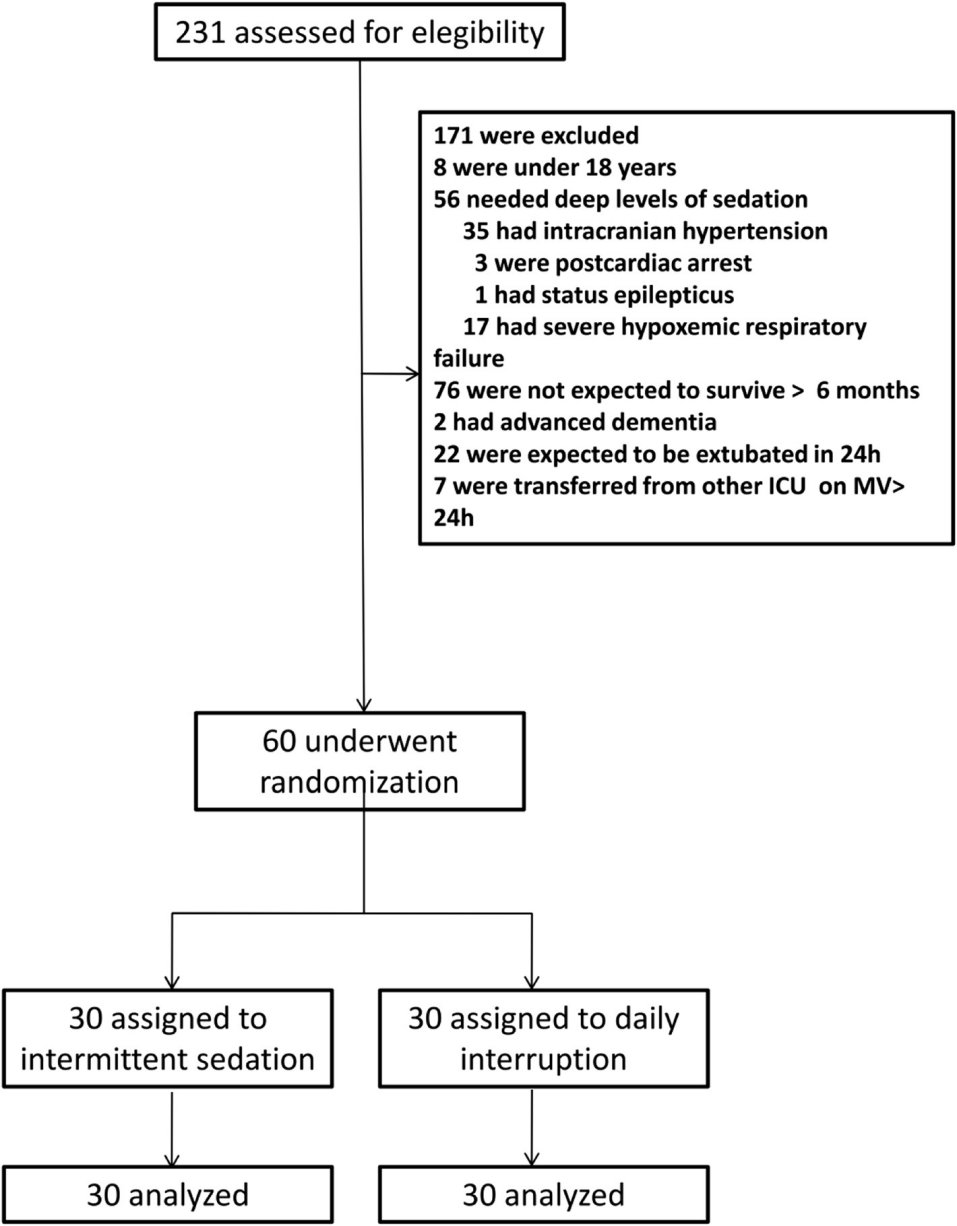
Assessment and randomization.

Two hundred and thirty-one patients were assessed for eligibility of whom 60 were included in the trial (Figure 1). Thirty patients (50%) were female, and median age was 49.5 (38.5, 58.75) years. Median APACHE II was 19 (16, 26.5). Emergency department and wards were the main sources of admission (29 (48.3%) and 26 (43.3%) patients, respectively). The main reasons for intubation were ARDS (n = 17, 28.3%), sepsis (n = 15, 25%) and pneumonia (n = 14, 23.2%) (Table 1). Twenty patients (66%) in each arm were enrolled less than 24 hours and all patients were enrolled 48 hours after starting mechanical ventilation.

**Table 1:** Baseline characteristics on admission to the ICU

**Characteristics**	**Intermittent sedation (n = 30)**	**Daily interruption (n = 30)**
Age (years)	47 (33,58)	51 (46,59)
Female sex, n (%)	13 (43)	17 (57)
Weight (kg)	61 (53,66)	57 (50,71)
APACHE II	22 (18,28)	18 (15.5,25)
PaO_2_/FiO_2_ ratio (mmHg)	178 (130,283)	160 (93,259)
Source of admission		
Emergency department, n (%)	15 (50)	14 (47)
Wards, n (%)	11 (37)	15 (50)
Surgical room, n (%)	3 (10)	1 (3)
Other ICU, n (%)	1 (3)	0 (0)
Diagnosis at admission		
Respiratory failure, n (%)	17 (57)	19 (63)
Sepsis syndromes, n (%)	12 (40)	9 (30)
Cardiogenic shock, n (%)	1 (3)	2 (7)
Reason for intubation		
ARDS, n (%)	9 (30)	8 (27)
Sepsis syndromes, n (%)	8 (27)	7 (23)
Pneumonia, n (%)	5 (16)	9 (30)
Acute pulmonary edema, n (%)	3 (10)	1 (3)
Other, n (%)	4 (13)	4 (13)

APACHE II denotes acute physiological and chronic health score.

ARDS denotes acute respiratory distress syndrome.

**Figure 2:**
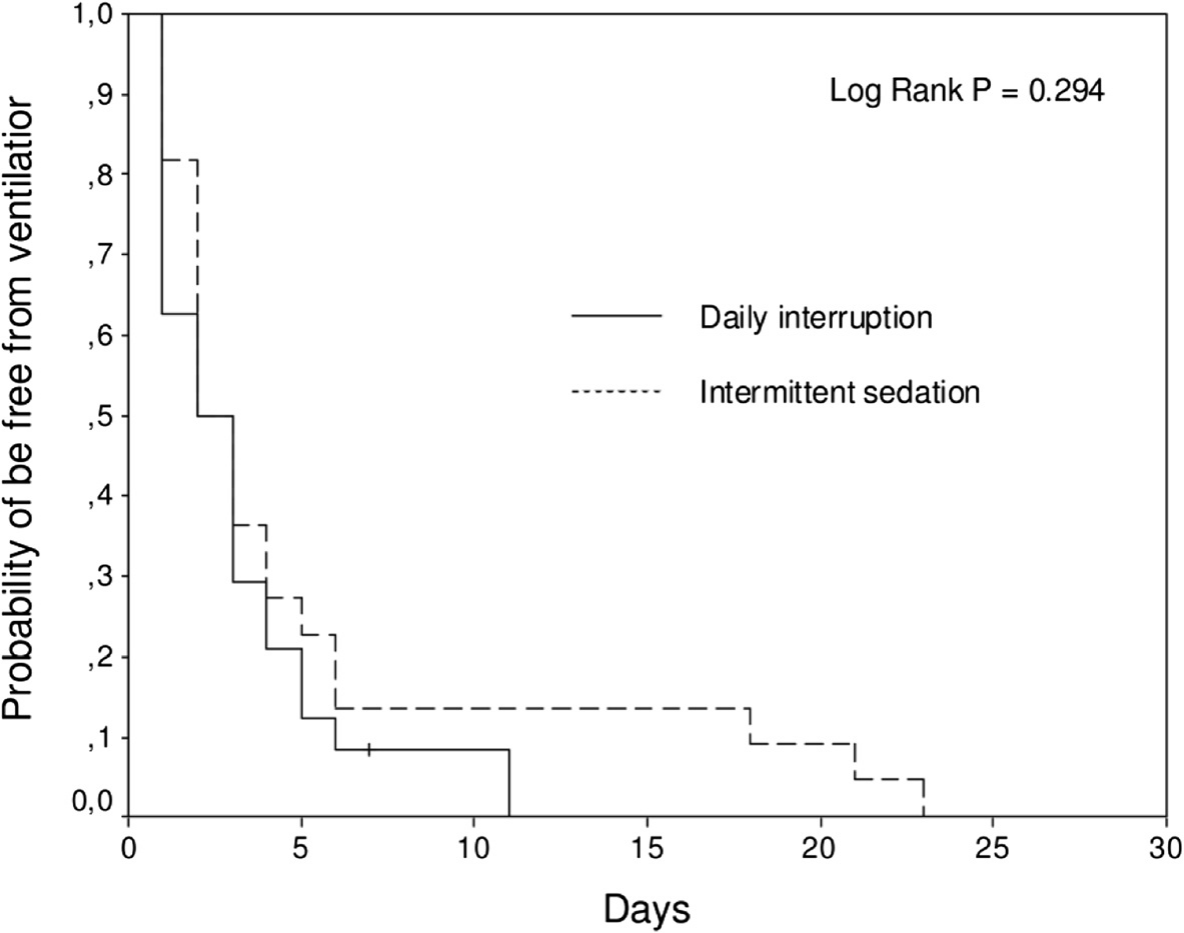
Kaplan-Meier plot of length of mechanical ventilation.

No difference was observed between the intermittent sedation and daily interruption groups in the number of ventilator-free days from intubation to day 28. ICU and hospital mortalities were also similar between the two groups. Incidence of delirium and number of days free of delirium or coma during the seven first days in ICU were also not different. ICU and hospital length-of-stay were similar between the two groups (Table 2). The probability of being alive and free of mechanical ventilation in 28 days was also the same between the two groups (Figure 2).

**Table 2:** Outcome and monitoring data of patients

**Outcomes**	**Intermittent sedation (n = 30)**	**Daily interruption (n = 30)**	***P-*** **value**
Ventilator-free days in 28 days, days	25 (21, 27)	24 (0, 26)	0.160
ICU mortality, n (%)	7 (23)	12 (40)	0.165
Hospital mortality, n (%)	9 (30)	13 (43.3)	0.284
Delirium, n (%)	12 (40)	9 (30)	0.472
Delirium or coma-free days, days	6 (1,7)	7 (3, 7)	0.514
Median SAS	3.6 (3.4, 4.0)	3.2 (2.6, 3.7)	0.035
Percentage of time on target SAS, (%)	75 (50, 100)	63 (42, 95)	0.624
ICU length of stay, days	11 (6, 16)	8 (5, 19)	0.595
Hospital length of stay, days	22 (13, 38)	15 (9, 28)	0.099
Reintubation, n (%)	1 (3)	4 (13)	0.161
Self-extubation, n (%)	2 (7)	1 (3)	0.514
Accidental removal of catheters, n (%)	1 (3)	2 (7)	0.554
Tracheostomy, n (%)	1 (3)	1 (3)	1.000
**Total drug dose per patient**			
Fentanyl, μg	300 (100, 1,520)	1,500 (520, 4,215)	0.004
Midazolam, mg	0 (0.0,0.5)	45 (0,201)	< 0.001
Propofol, mg	0 (0.0,0.0)	0 (0.0,0.0)	0.799
Haloperidol, mg	0 (0.0,0.0)	0 (0.0,0.0)	0.280

There was no significant difference between the time that patients stayed in the target SAS. However, the intermittent sedation group had a higher SAS than the daily interruption group (median 3.6 (3.4, 4.0) versus 3.2 (2.6, 3.7); *P* = 0.035) (Table 2). Self-extubation occurred in one patient in the intermittent sedation group and in two patients in the daily interruption group. Out of these patients, one in the intermittent sedation and one patient in the daily interruption group had to be re-intubated. After extubation, re-intubation occurred in 8% of the patients, and was more common in the daily interruption group (four versus one patient), although not statistically significant. One patient in each group had an accidental removal of catheters, and tracheotomy was also performed on one patient in each group (Table 2).

**Figure 3:**
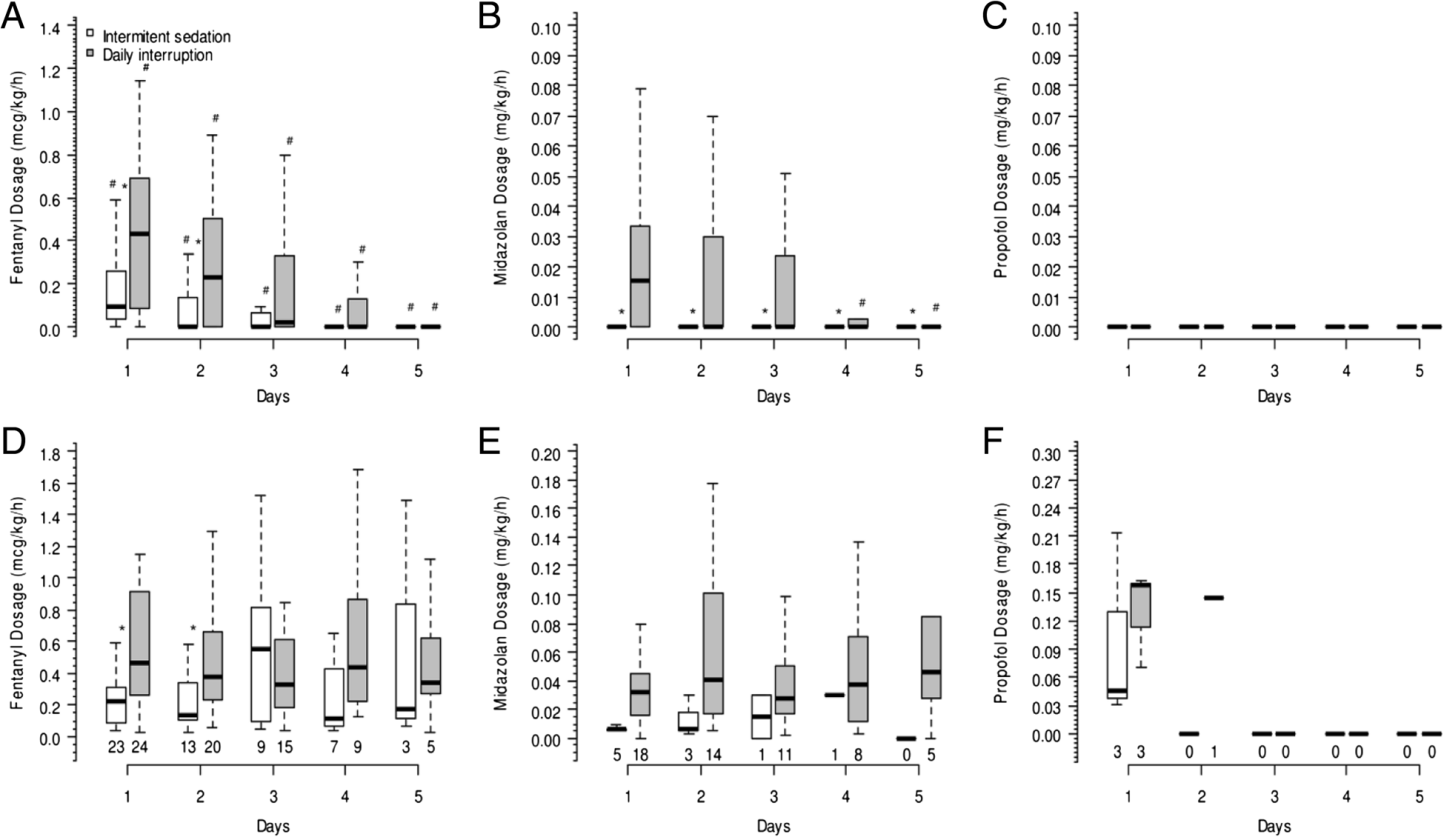
**Drug dosages of both groups up to the fifth day after mechanical ventilation initiation.** Panel **(A)** shows the median dosage of fentanyl of all 60 patients (*P* < 0.001 for time interaction and *P* = 0.015 for group interaction); panel **(B)** shows the median dosage of midazolam of all 60 patients (*P* = 0.025 for time interaction and *P* = 0.002 for group interaction); panel **(C)** shows the median dosage of propofol of all 60 patients (*P* = 0.124 for time interaction and *P* = 0.549 for group interaction); panel **(D)** shows the median dosage of fentanyl of patients who used fentanyl in at least one day during the first five days after ICU admission (*P* = 0.729 for time interaction and *P* = 0.021 for group interaction); panel **(E)** shows the median dosage of midazolam of patients who used midazolam in at least one day during the first five days after ICU admission (*P* = 0.299 for time interaction and *P* = 0.169 for group interaction); and panel **(F)** shows the median dosage of propofol of patients who used propofol in at least one day during the first five days after ICU admission (*P* = 0.907 for time interaction and *P* = 0.641 for group interaction).

Midazolam was given more frequently to patients in the daily interruption group (21 (70%) versus 7 (23%) patients, *P* < 0.001). Five patients (16.7%) in both groups received propofol (*P* = 1.0). Continuous infusion of fentanyl was used in 25 (83.3%) patients in the intermittent sedation group and in 26 (86.7%) patients in the daily interruption group (*P* = 0.718). There was no significant difference in total doses of propofol and haloperidol (Table 2). Fentanyl and midazolam total dosages were higher in the daily interruption group (Table 2, Figure 3). The dosage of fentanyl was decreased from day 1 to 5 in both groups. However, the fentanyl dosage was higher in the daily interruption group when taking into account all patients (Figure 3A). Furthermore, the dosage of the drug among patients who received fentanyl was also higher in the daily interruption group (Figure 3D). Midazolam was rarely used in the intermittent sedation group, but whenever used, it was administered in lower dosages.

**Figure 4:**
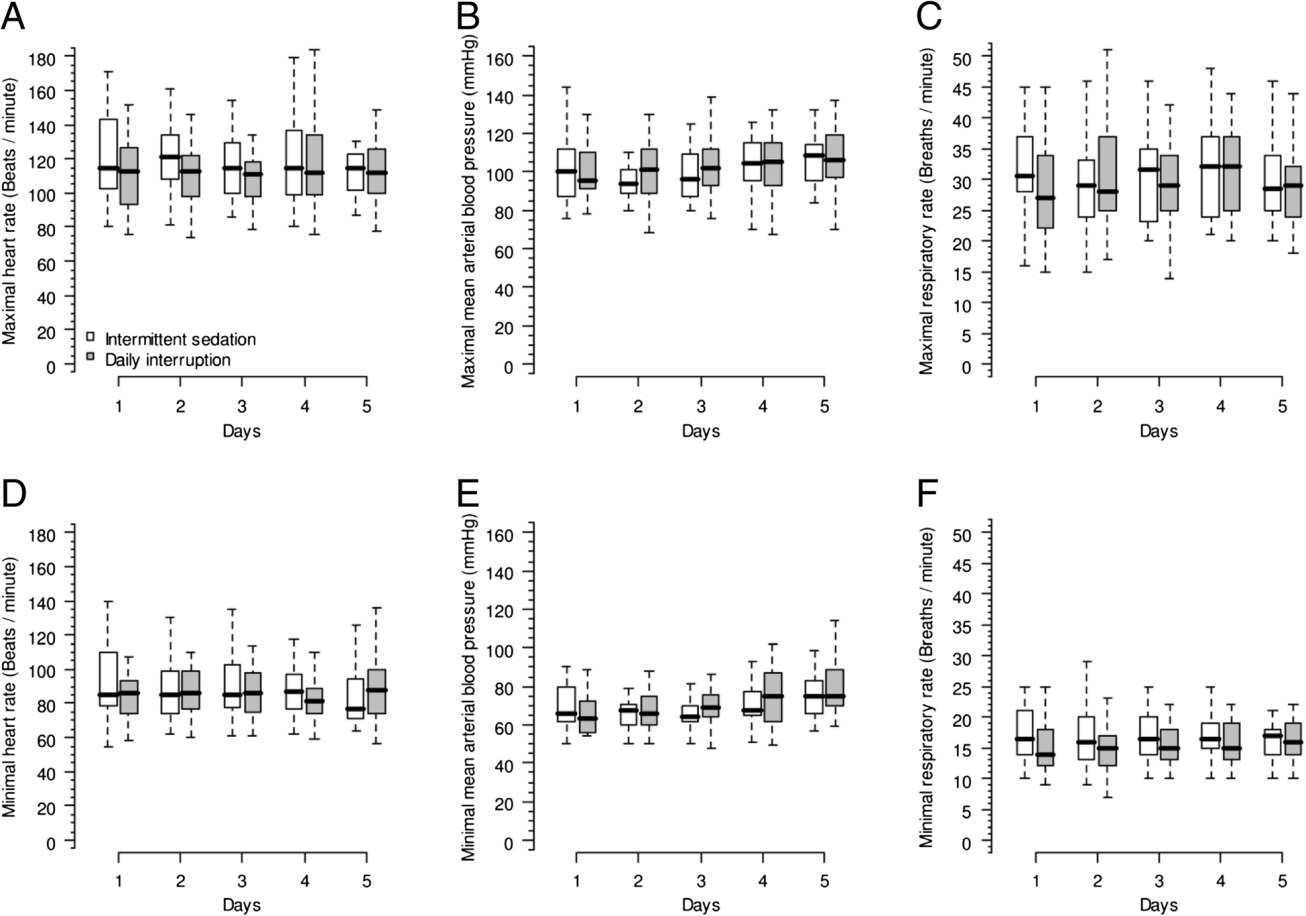
**Physiologic variables of both groups up to the fifth day after mechanical ventilation initiation.** Panels **(A)** (mixed model fixed effects *P* = 0.412 for time interaction and *P* = 0.202 for group interaction) and **(D)** (mixed model fixed effects *P* = 0.047 for time interaction and *P* = 0.608 for group interaction) show the maximal and minimal heart rate; panels **(B)** (mixed model fixed effects *P* < 0.001 for time interaction and *P* = 0.566 for group interaction) and **(E)** (mixed model fixed effects *P* < 0.001 for time interaction and *P* = 0.524 for group interaction) show the mean arterial blood pressure; and panels **(C)** (mixed model fixed effects *P* = 0.462 for time interaction and *P* = 0.801 for group interaction) and **(F)** (mixed model fixed effects *P* = 0.991 for time interaction and *P* = 0.259 for group interaction) show the respiratory rate.

Evolution of heart rate, mean arterial pressure and respiratory rate during the first five days of the study were not different between the two groups (Figure 4). There were no differences in the use of norepinephrine and dobutamine between the groups. Fluid balances were progressively decreased during the first five days compared to the first day in both groups. NAS values were not different between the two groups during the first five days of the study. However, NAS reduced similarly in both groups on days 2 to 5 compared to day 1. Total SOFA scores followed the same tendency (Table 3).

**Table 3:** Data about monitoring, nurses workload, ventilatory and hemodynamic support during the first five days of mechanical ventilation admission

**Ventilatory support**	**Group**	**Day 1**	**Day 2**	**Day 3**	**Day 4**	**Day 5**	***P*-value**
Patients alive, n (%)	Intermittent sedation	30 (100)	30 (100)	28 (93)	25 (84)	24 (80)	0.999
	Daily interruption	30 (100)	28 (93)	26 (87)	25 (84)	24 (80)	
Patients on mechanical ventilation, n (%)	Intermittent sedation	30 (100)	19 (63)	17 (61)	10 (40)	7 (29)	0.858
	Daily interruption	30 (100)	24 (86)	15 (58)	12 (48)	11 (46)	
Pressure support, n (%)	Intermittent sedation	28 (93)	18 (95)	16 (94)	10 (100)	7 (100)	0.763
	Daily interruption	23 (77)	20 (84)	11 (73)	12 (100)	9 (82)	
Controlled mode, n (%)	Intermittent sedation	2 (7)	7 (5)	1 (6)	0 (0)	0 (0)	0.686
	Daily interruption	7 (23)	4 (16)	4 (27)	0 (0)	2 (18)	
**Hemodynamic support**							
Norepinephrine, n (%)	Intermittent sedation	18 (60)	13 (43)	11 (39)	2 (8)	1 (4)	0.405
	Daily interruption	10 (33)	6 (21)	2 (8)	2 (8)	2 (8)	
Dobutamine, n (%)	Intermittent sedation	13 (43)	12 (40)	11 (39)	7 (28)	3 (13)	0.917
	Daily interruption	4 (13)	4 (14)	3 (12)	2 (8)	0 (0)	
**Monitoring**							
Fluid balance, mL	Intermittent sedation	935 (323, 2,134)	960 (421, 2,189)	−21 (-671, 1,028)^d^	237 (-665, 936)^d^	−187 (-852, 1,336)^d^	< 0.001^c^
	Daily interruption	743 (112, 1,836)	190 (-705, 1,172)^d^	−289 (-1,143, 743)^d^	−106 (-804, 849)^d^	180 (-1,092, 1,350)^d^	0.449^b^
Nursing activity score^a^	Intermittent sedation	54 (49,56)	48 (45, 52)^d^	48 (42, 52)^d^	43 (38, 45)^d^	39 (34, 46)^d^	< 0.001^c^
	Daily interruption	53 (51,54)	45 (43, 50)^d^	45 (40, 52)^d^	43 (37, 45)^d^	38 (34, 44)^d^	0.292^b^
Total SOFA score	Intermittent sedation	9 (7,12)	8 (4, 11)	9 (2, 12)	7 (2, 10)^d^	7 (2, 0)^d^	< 0.001^c^
	Daily interruption	7 (4,10)	7 (4, 10)	5 (3, 8)^d^	4 (3, 9)^d^	4 (3, 11)^d^	0.545^b^

^a^Time spent for the care of a given patient within a shift.

^b^Mixed model fixed effects-time interaction.

^c^Mixed model fixed effects-group interaction.

^d^*P* < 0.05 versus first day.

SOFA, sequential organ failure assessment score.

**Figure 5:**
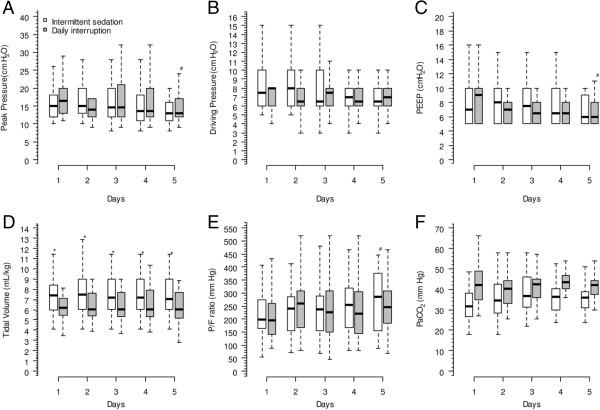
**Respiratory variables of both groups up to the fifth day after mechanical ventilation initiation.** Panel **(A)** shows the peak pressure of all patients (*P* = 0.002 for time interaction and *P* = 0.549 for group interaction); panel **(B)** shows the driving pressure of all patients (*P* = 0.456 for time interaction and *P* = 0.549 for group interaction); panel **(C)** shows the median dosage of propofol of all patients (*P* = 0.001 for time interaction and *P* = 0.929 for group interaction); panel **(D)** shows the driving pressure of all patients (*P* = 0.549 for time interaction and *P* = 0.001 for group interaction); panel **(E)** shows the PaO_2_/FiO_2_ (P/F) ratio of the patients (*P* = 0.002 for time interaction and *P* = 0.793 for group interaction); and panel **(F)** shows the PaCO_2_ of all patients (*P* = 0.151 for time interaction and *P* = 0.334 for group interaction). *P < 0.05 versus intermittent sedation group, Mann-Whitney *post hoc* test. #*P* < 0.05 versus first day of the same group, Mann-Whitney *post hoc* test.

Pressure support was the chosen ventilator mode in a larger number of the patients during the first five days in both groups (Table 3). Ventilation variables were not different between groups in relation to peak pressure, driving pressure, PEEP and PaCO_2_. Tidal volumes were higher in intermittent sedation group during all five days (Figure 5).

After six months of ICU discharge, only 21 of the 38 patients who were discharged alive from the hospital could be evaluated with the IES. Two in the intermittent sedation and one in the daily interruption group died after hospital discharge, and 14 could not be contacted. There were 13 patients in the intermittent sedation and 8 patients in the daily interruption group who answered the IES. Median scores were higher in daily interruption sedation group, what indicates higher levels of psychological distress, although not statistically different (22 (8, 31) versus 16 (4, 34), *P* = 0.750).

## Discussion

To the best of our knowledge, our study was the first randomized controlled trial aiming to compare two sedation strategies in a developing country with a low nursing staff level. Although it was underpowered to show any difference in duration of mechanical ventilation between intermittent sedation and daily interruption of sedation, it suggests that both strategies seem to be safe in terms of complications, and are not different regarding nurse workload - two very important issues in units with profiles similar to ours. Both strategies are also secure in terms of psychological distress six months after hospital discharge. These results indicate that lighter sedation approaches may be feasible and safe even in lower nursing staff level ICUs.

Deep sedation is a huge problem in critically ill patients on mechanical ventilation. Continuous sedation has been associated with prolonged times on mechanical ventilation for more than a decade [2]. Since then, many studies have shown that protocols of sedation which allow patients to be awake or easily arousable are associated with fewer days on mechanical ventilation and in the ICU [8,18]. However, in ICUs with very high staff levels, protocols of sedation may not be necessary [19].

Daily interruption of sedative infusions was another strategy that has shown to be effective in reducing timing on mechanical ventilation [6,7]. It is not associated with excessive complications rate as compared to the usual practice [20], and it is safe in terms of psychological distress after hospital discharge [5,21]. A systematic review suggested that daily interruption of sedatives had a higher level of evidence than sedation protocols [22], but comparisons between the two are scarce.

There have been five studies before ours comparing daily interruption of sedatives with a specified sedation protocol. A Boston study included 74 medical patients and compared daily interruption with a sedation protocol. In that study, daily interruption of sedatives was associated with a prolonged duration of mechanical ventilation (almost three days), ICU length of stay (seven days), and length of hospital stay (eleven days) [23]. A pilot Canadian trial compared a sedation protocol with the same sedation protocol associated with daily interruption of sedative infusions in 65 patients. They found no difference between the two approaches in terms of duration of mechanical ventilation or ICU length of stay [24]. A subsequent multicenter trial based on this pilot was conducted in Canada and the United States with 423 patients, and also evidenced no difference in time to successful extubation, ICU and hospital length of stay [25]. A Greek trial included 97 patients, and also showed no difference in duration of mechanical ventilation, ICU and hospital stay between daily interruption of sedative infusions and a practice of early awakening of patients [26]. A recent Danish study with 113 patients suggested that a protocol of no-sedation, that kept patients with scheduled infusions of morphine and instituted continuous infusion of propofol for short periods of time, if necessary, was associated with more days without mechanical ventilation and shorter ICU and hospital stays [9].

Except for the Canadian trials, all of them were single-center trials. Therefore, they expressed local practices, and their results can only be compared and externally applied with some caution. The Boston study [23] targeted a deeper level of sedation than all other studies and the original description of daily interruption of sedative infusions [6]. Also, this is the only study which included only medical patients. Consequently, their results may be very different from the others. The Canadian trials [24,25] had the exact same target as ours, but they studied continuous infusion of sedatives on both arms. The Greek trial [26] had a target of sedation similar to ours, although their protocol used continuous infusion of sedatives and remifentanyl as the analgesic drug. It seems that the study that is most similar to ours is the Danish one [9]. Like ours, that study allowed patients to be maintained without any sedative, used propofol or midazolam in the daily interruption arm (although in a different fashion), but kept patients with scheduled morphine infusions, while we used fentanyl as analgesic. The Danish study ICU also had a higher nurse-to-patient ratio. However, their patients had fewer days free of mechanical ventilation. We may postulate that the impact of reducing sedative agents appears only when patients need to be on mechanical ventilation for longer periods of time. Nevertheless, we have shown that a strategy of intermittent sedation is feasible even in a lower staff level ICU.

Recently, it has been shown that not only are deeper levels of sedation associated with worse outcomes, but also early deep sedation (that is at the first 48 hours after admission) is associated with delayed extubation and higher mortality [3,27]. Most clinical trials aiming to reduce the burden of sedation only enrolled patients after 48 hours of mechanical ventilation [7,18,28]. In our trial, both interventions started very early, since all patients were randomized in less than 48 hours, and we used a very small amount of sedatives in both arms compared to previous [6,7,23-25], but not all studies [9]. Like ours, a recent pilot clinical trial has shown the feasibility of achieving an early lighter sedation [29].

It has been thought that deeper sedation levels were associated with higher levels of comfort. However, recent findings from clinical trials were not able to demonstrate those ideas. The greater cardiovascular safety associated with deeper sedation is also speculative. The daily sedative interruption is not associated with higher cardiovascular events in high risk patients [30]. In this trial, the physiological variables were similar in both groups, a fact that points out the safety of light sedation levels in mixed critically ill patients.

In our study, tidal volumes were higher in the intermittent sedation group. Although we excluded patients with severe hypoxemic respiratory failure, it is worth noting that this strategy may be harmful for these patients, since tidal volumes would not be strictly controlled [31].

Nurse workload was similar in both groups and decreased during the first five days of mechanical ventilation (Table 3). By contrast, in the Danish no-sedation trial, the awake patients needed an extra person to take care of them [9]. Although our data does not show significant increase in workload, it is advisable to exercise caution before widely adapting this sedation strategy in a lower staff ICU. As we mentioned before, we began the intermittent-sedation strategy in 2006. Perhaps, at that time, this approach may have been associated with a higher nurse workload, but then, an adaptive process took place. Nevertheless, in this study, lighter sedation was not associated with a number of adverse events that may be commonly related to lower staffing levels, such as self-extubation or accidental removal of catheters. More importantly, these adverse events were not different from those presented in other studies [9,25].

Lighter levels of sedation have been associated with safety [21,32] or even better psychological outcomes when compared to deeper sedation levels [5,18]. Our study, like the Danish study [32], suggests that intermittent sedation is not associated with worse psychological outcomes than daily interruption of sedative infusion. However, our conclusions are derived from a very small sample of patients since we were not able to reach a large number of patients who were discharged alive from the hospital.

We believe our trial has some strengths. First, it has shown that lighter sedation levels are feasible and safe even in a lower nursing staff ICU. Second, we studied two approaches which prioritize pain assessment and management. This is in accordance with what is recommended in international guidelines [33]. Therefore, it seems that it is possible to adopt light sedation strategies even in low nursing staff ICUs in order to reduce the duration of mechanical ventilation.

On the other hand, our study also has several limitations. First, it was conducted in a single ICU in a teaching hospital. Therefore, our results may not be generalizable to services with different profiles. Second, and most importantly, we were not able to reach the target sample. Nevertheless, as commented before, it would be necessary to analyze a larger sample than it was initially calculated in order to find a statistically significant result that could still not be clinically meaningful. Third, we hypothesized that intermittent sedation would increase the number of mechanical ventilation-free days. However, since both strategies reached very close levels of sedation, a non-inferiority design would probably have been a better approach to address this question. Fourth, we did not use dexmedetomidine as a sedative agent, which has been associated with positive findings when compared to benzodiazepines [28,34], but not with propofol [28].

## Conclusions

Intermittent sedation and daily interruption of sedation were feasible strategies in order to reduce the burden of sedation overuse in a low nursing staff ICU. Both approaches were safe in terms of complications, such as accidental extubation and removal of catheters, and in terms of psychological distress after hospital discharge. Intermittent sedation was associated with lower sedatives and opioids doses, which may reduce costs in ICUs in developing countries.

## Abbreviations

APaCHE: Acute Physiological and Chronic Health Score; ARDS: Acute Respiratory Distress Syndrome; CAM-ICU: Confusion Assessment Method in ICU; IES: Impact of Event Scale; NAS: Nurse Activity Score; SAS: Sedation-Agitation Scale; SOFA: Sequential Organ Failure Assessment.

## Competing interests

The authors declare that they have no competing interests.

## Authors’ contributions

APNJ participated in the design of the study, collection, analysis and interpretation of the data. MP participated in the conception and design of the study, analysis and interpretation of the data, and critical revision of the manuscript. Both authors approved the final manuscript.
